# Reverse Transcriptase Mechanism of Somatic Hypermutation: 60 Years of Clonal Selection Theory

**DOI:** 10.3389/fimmu.2017.01611

**Published:** 2017-11-23

**Authors:** Edward J. Steele

**Affiliations:** ^1^CYO’Connor ERADE Village Foundation Inc., Piara Waters, WA, Australia

**Keywords:** somatic hypermutation, strand-biased mutations, DNA polymerase-η, A-to-I RNA and DNA editing, RNA exosome, AID-deaminase, reverse transcription

## Abstract

The evidence for the reverse transcriptase mechanism of somatic hypermutation is substantial and multifactorial. In this 60th anniversary year of the publication of Sir MacFarlane Burnet’s Clonal Selection Theory, the evidence is briefly reviewed and updated.

## Overview

**Figure 1:**
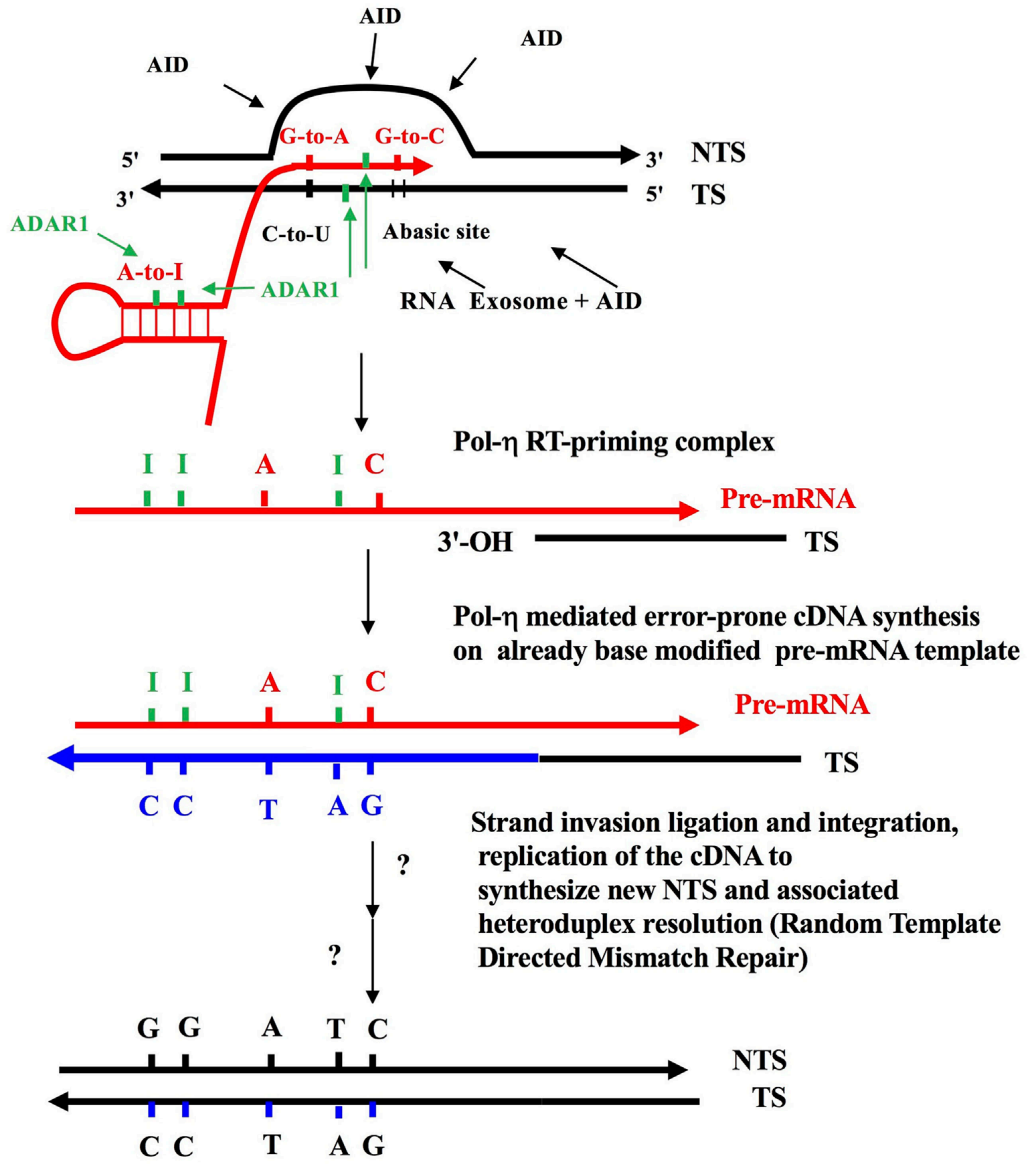
The reverse transcriptase mechanism of somatic hypermutation (SHM). Some elements of this figure have appeared before, and this figure *in toto* is a modified combination of parts from Figure 1 in Lindley and Steele (10), as well as from figures in Steele (9, 11) and Steele and Lindley (14). This is also an adaptation of the target site reverse transcription process of Luan et al. (66). Shown is an RNA Polymerase II-generated Transcription Bubble with C-site and A-site substrate deamination events by AID and ADAR proteins, which generates the strand-biased mutation signatures—A-to-G, G-to-A, G-to-T, and G-to-C (9, 11, 14). DNA strands shown by black lines; pre-mRNA as red lines; cDNA strands as thick blue lines due to DNA polymerase η (59). Green bars are Inosines. Shown also is the action of the RNA exosome (64) allowing access of AID deaminase to cytosines on the transcribed strand (TS). The ssDNA regions on the displaced non-transcribed strand (NTS) are established targets of AID action (53–56). Note that DNA mutations are first introduced as AID-mediated C-to-U, followed by excision of uracils by DNA glycosylase (UNG), which creates Abasic sites in the TS (these can mature into single strand nicks with 3′-OH ends via the action of AP endonuclease). These template Uracil and Abasic sites can be copied into pre-mRNA by RNA Pol II generating G-to-A and G-to-C modifications as shown (67). Following target site reverse transcription (66), this results in G-to-A and G-to-C mutations in the NTS, in a strand biased manner (9–11, 14). Separately at WA targets in nascent dsRNA substrates, adenosine-to-inosine (A-to-I) RNA editing events, mediated by ADAR1 deaminase, are copied back into DNA by reverse transcription via Pol-η (59). In theory, ADARs can also deaminate the RNA and DNA moieties in the RNA: DNA hybrid (14, 15). The strand invasion and integration of newly synthesized cDNA TS, as well as random-template mismatch repair (68) are hypothesized additional steps (not shown here). In short, RNA Pol II introduces modifications in the Ig pre-mRNA as it copies TS DNA with AID lesions and this is coupled to A-to-I in dsRNA stem-loops near the transcription bubble (62) as well as in RNA:DNA hybrids within the bubble (14, 15). Next, a RT-priming substrate is formed when the nicked TS strand with an exposed 3′-OH end anneals with the base modified pre-mRNA copying template allowing cDNA synthesis by Y Family translesion DNA polymerase-η (48), now acting in its reverse transcriptase mode (59). These 3′-OH annealed priming sites could arise due to excisions at previous AID-mediated Abasic sites. Alternatively, they could arise due to an endonuclease excision associated with the MSH2-MSH6 heterodimer engaging a U:G mispaired lesion (61). Shown is an A-to-T transversion generated at the RT step at a template Inosine. ADAR, Adenosine Deaminase that acts on RNA; AP, an Abasic, or apurinic/apyrimidinic, site; APOBEC family, generic abbreviation for the dC-to-dU deaminase family of which AID is a member (e.g., APOBEC1; APOBEC3 A, B, C, D, F, G, H); AID, activation induced cytidine deaminase causing C-to-U lesions at WRCY/RGYW C-site motifs in ssDNA; W, A, or U/T; WA-site, target motif for ADAR deaminase including DNA polymerase-η error prone incorporation *in vitro* (50, 51); Y, pyrimidines T/U or C.; R, purine A or G.

The molecular mechanism underlying somatic hypermutation (SHM) of rearranged immunoglobulin (Ig) genes (V[D]J) has been controversial for some time. Although the process of DNA deamination has dominated discussion in recent years, insufficient attention has been paid to a mechanism based on reverse transcription. One reason therefore for writing this *Perspective* is to counter balance a widely held view in the Ig SHM field that all relevant studies on the molecular mechanism deal only with the “DNA Deamination Model” which ended in complete consensus over 10 years ago sometime between 2004 and 2007 [Table 1 and Ref. (1) in particular]. The other is a personal tribute, in this anniversary year, to the founder of modern immunology, Sir Macfarlane Burnet. It is now 60 years since the publication of the first iteration of “The Clonal Selection Theory of Acquired Immunity” (2), the foundation stone of modern immunology. It was fully expounded in his 1959 book (3) where the main idea was clonal antigenic selection from a pre-existing diverse antibody repertoire from which somatic mutations might emerge as “forbidden” anti-self clones. Joshua Lederberg then gave the concept sharp molecular focus (4) as did Melvin Cohn and colleagues (5–7). Alastair Cunningham’s concept of “clonal variation around a theme” placed antigen-driven SHM firmly within the context of expanding B lymphocyte clones (8). Somatic mutation of Ig variable region genes has therefore been part and parcel of Burnet’s clonal selection concept since its inception and is central to a rational understanding of immunological diversification, self-tolerance and the emergence of cancer. We now have a very good idea of the molecular mechanism of SHM. I have chosen to fit this scientific progress within 60 key publications since the late 1950s (Table 1). The most plausible central molecular mechanism of Ig SHM, that fits with and explains all the evidence (9–11) is based on “Reverse Transcription” of the base-modified Ig pre-mRNA (Figure 1). That is, error-prone reverse transcription, by DNA Polymerase-η, of the Ig pre-mRNA template intermediate at rearranged V[D]J gene somatic loci. The Ig pre-mRNA encoding the V[D]J region is copied off the transcribed DNA strand carrying prior AID C-to-U deamination lesions (Uracils and Abasic sites), and it also accumulates ADAR-deaminase mediated RNA editing A-to-I modifications. This already base-modified pre-mRNA sequence is then copied back to the B lymphocyte genomic DNA and integrated at the rearranged V[D]J site (concurrent with antigen-mediated selection of Ig receptor bearing B lymphocytes, Centrocytes, in the Germinal Center). This is essentially the “Reverse Transcriptase Mechanism” which Jeff Pollard and I first published 30 years ago (12). The mechanistic steps, many logical, are clearly outlined in Figure 1, which shows that the characteristic A >> T and G >> C strand bias-generating mutagenic activity is firmly focused on the nascent RNA intermediate in the context of the Transcription Bubble (9–11, 13, 14). Recent publications should be consulted for further definitive ADAR A-to-I editing of both RNA and DNA moieties at RNA:DNA hybrids within Transcription Bubbles (11, 14, 15). Not only is it important to understand the correct molecular mechanism of SHM for cancer diagnosis and detection (16, 17) but also to the current efforts to better understand (18, 19) the origin of Ig diversity involving the mechanism of evolution of the sets germline V segments and the long IGHV and IGLV haplotypes in individual human beings (20, 21).

**Table 1:** History of somatic hypermutation (SHM): developments relevant to the reverse transcriptase mechanism.

Year	Author	Main development-discovery-concept	Reference
1957–1959	Burnet	Large repertoire of antibodies each lymphocyte produces one specific antibody	(2)
1959	Lederberg	Somatic mutation explicit in lymphocyte development and Ab diversity	(4)
1962	Fleishman et al.	Amino acid variation in N -terminal regions of V or antigen binding regions	(22)
1966	Brenner and Milstein	Model: V region specific nicking and error prone repair—“SHM”	(23)
1967	Smithies	Somatic “Master-> Slave” Gene Recombination model Ab diversity	(24)
1967	Edeleman and Gally	Somatic recombination between duplicated V genes model Ab diversity	(25)
1968	Cohn	Molecular biology of expectation—rationale for SHM and response to unexpected	(5)
1970	Weigert et al.	Somatic variability in Lambda light chain V region protein sequences	(6)
1970	Wu and Kabat	Hypervariable regions coincide with and define antigen contact regions	(26)
1974	Cunningham	The generation of antibody diversity after antigen	(8)
1974	Cohn	Somatic mutation explanation for Ab diversity clearly laid out	(7)
1976	Tonegawa and Steinberg	DNA V gene counting confirms somatic mutation at molecular level in V lambda	(27)
1977	Tonegawa et al.	DNA V gene counting confirms somatic mutation at molecular level in V lambda	(28)
1981	Gearhart et al.	SHM of the TEPC15 VH rearranged gene *in vivo*	(29)
1981	Bothwell et al.	SHM to the VH186.2 VH rearranged gene *in vivo*	(30)
1981	Seising and Storb	SHM of the MOPC167 VK rearranged gene *in vivo*	(31)
1982	Gearhart	SHM in Rearranged (VDJ) Variable Region Genes *In vivo*	(32)
1983	Gearhart and Bogenhagen	Somatic mutations occur in the 5′ and 3′ non-coding regions around VDJ genes	(33)
1985	Berek and Milstein	Use of hybridoma technique to sample somatic V[D]J mutant generation *in vivo*	(34)
1986	Cumano and Rajewsky	Further use hybridoma technique to sample somatic VDJ mutants *in vivo*	(35)
1987	Steele and Pollard	Model: the reverse transcriptase mechanism of SHM	(12)
1987	Golding et al.	First hint of strand biases in SHM patterns viz. A > G *versus* T > C	(36)
1990	Both et al.	Defining the 5′ and 3′ boundaries of SHM at VDJ genes	(37)
1990	Lebecque and Gearhart	Defining 5′ and 3′ boundaries of SHM at VDJ genes	(38)
1991–1996	Rogozin et al.	Identification RGYW/WRCY and WA hotspots in SHM data	(39, 40)
1992	Steele et al.	Defining the asymmetrical 5′ to 3′ somatic mutation distribution around V[D]J genes	(41)
1993	Betz et al.	Defining the mutational hot spots across mutated V[D]J transgenes genes	(42)
1995	Yelamos et al.	Any non-lg sequences parked between Promotor and J-C intron somatically mutates	(43)
1996	Peters and Storb	Strong evidence that transcription of VDJ target regions allows somatic mutation	(44)
1995–1998	Blanden et al.	The SHM signature is written into the germline V segment array	(18)
1998	Milstein et al.	Both DNA strands targeted for G:C and A:T mutations in SHM	(45)
1998	Fukita et al.	Strong correlative evidence that transcription of VDJ allows somatic mutation	(46)
1998	Rada et al.	In MSH2-deficient mice mutations are G:C focused suggesting two stages SHM	(47)
1999	Masutani et al.	Discovery of DNA Polymerase -eta and Y family translesion polymerases	(48)
2000	Muramatsu et al.	AID discovered—required to intiate SHM and Ig Class Switch Recombination	(49)
2001–2002	Rogozin et al.; Pavlov et al.	Error-prone DNA Polymerase eta SHM spectrum correlates with WA hotspots	(50, 51)
2001	Zeng et al.	DNA Polymerase eta is the A:T mutator in SHM in humans	(52)
2002–2004	Neuberger et al.	Definitive evidence that AID is a direct DNA C-to-U deaminase of the APOBEC family	(1)
2003	Bransteitter et al.	AID deaminates C > U on ssDNA—targets displaced strand Transcription Bubble	(53)
2003	Chaudhuri et al.	AID deaminates C > U on ssDNA—targets displaced strand Transcription Bubble	(54)
2003	Dickerson et al.	AID deaminates C > U on ssDNA—targets displaced strand Transcription Bubble	(55)
2004	Chaudhuri et al.	AID deaminates C > U on ssDNA—targets displaced strand Transcription Bubble	(56)
2004	Shen and Storb	AID targets both strands at Transcription Bubbles during transcription VDJ	(57)
2004	Rada et al.	MSH2-MSH6 -/-and Uracil DNA Glycosylase -/-define G:C and A:T mutation phases	(58)
2004	Franklin et al.	Human DNA Polymerase eta is an efficient reverse transcriptase, as are kapp, iota	(59)
2004	Steele et al.	First hint that A > G versus T > C strand bias involves an A > l RNA edited intermediate	(60)
2005	Wilson et al.	MSH2-MSH6 stimulates DNA polymerase eta, suggesting a role for A:T mutations	(61)
2006	Steele et al	Evidence WA > WG mutations correlate with the number nascent WA RNA stem loops	(62)
2007	Delbos et al.	Evidence that DNA Polymerase eta is the sole error-prone A:T SHM mutator *in vivo*	(63)
2009	Steele	SHM data 1984–2008 shows A»T, G»C strand biases explained by RNA/RT-model	(9)
2010–2013	Steele and Lindley; Lindley and Steele	A>>T, G>>T SHM strand biases evident in non-lg genes across all cancer exomes	(10, 13)
2011	Basu et al.	RNA exosome exposes ssDNA for AID on transcribed strand at Transcription Bubbles	(64)
2011	Maul et al.	AID generated Uracils physically located in the DNA of VDJ & Ig class switch regions	(65)
2013	Lindley	Codon-context targeted somatic mutation in cancer exomes	(16)
2016	Steele	Extant evidence supports the RNA/RT-based model and not the DNA-based model	(11)
2017	Zheng et al.	ADAR can directly edit both RNA and DNA A-sites in RNA:DNA hybrids	(15)
2017	Steele and Lindley	ADAR A > l Editing at RNA:DNA Hybrids is strong support for RNA/RT-based model	(14)

## Critical Focus on the RNA/RT-Mechanism

The author has comprehensively reviewed the detailed evidence for the reverse transcription-based mechanism of SHM in previous and current studies (9–11). However, as flagged at the start of this article, many immunology researchers describe the mechanism of Ig SHM as being via DNA Polymerase-η-mediated DNA lesion repair independent of pre-mRNA in the context of the AID-initiated “DNA Deamination Model.” It will be informative then to not only refer to these literatures but also summarize the evidence directly supporting an Ig pre-mRNA intermediate and reverse transcription, as summarized in Figure 1.

The alternative to the RNA/RT-based mechanism is the “DNA Deamination Model,” which is assumed to be coupled to direct DNA-based error-prone repair via translesion DNA polymerase-η acting solely by error-prone copying of DNA templates (50, 51) during gap-repair surrounding AID-generated lesions (Uracils, Abasic sites, ssDNA nicks), as outlined in detail by Neuberger and associates (1, 58), Gearhart and associates (61, 65), and many other laboratories (53–57, 63) published mainly in the period 2002–2011. Quite apart from all the data at odds or inconsistent with this alternative theory, there have been three direct published tests of the Reverse Transcriptase Mechanism since 2001, one study was inconclusive and two studies reported positive data directly consistent with the RNA/RT-based mechanism.

In the first direct test of the RT model, Sack et al. (69) treated immunized mice with retroviral RT inhibitors, AZT, ddC and determined mutation frequencies in the anti-NP response of the rearranged V_H_186.2 sequence from control and test mice and showed a systematic lowering of the somatic mutation frequency by about 33–35% in both test groups compared to the control [see Table 2 in Ref. (69)]. The authors however concluded that these retroviral RT inhibitors had no statistically significant effect (the *P* values were *P* = 0.056 and *P* = 0.069, respectively), thus claiming that “standard reverse transcription is not required for antibody V region hypermutation in the mouse” (69). This study and the conclusions drawn have been critically evaluated, and the present author considers that the data published in Sack et al. (69) have been misinterpreted (9, 11, 70).

In the next test, Franklin et al. [(59), Figure 1 and legend] showed that the sole known error-prone DNA polymerase involved in Ig SHM, DNA Polymerase-η (52, 63) is a very efficient reverse transcriptase: as indeed are human DNA Polymerases iota (-ι) and kappa (-κ) although less active than eta (-η).

Lastly Steele et al. (62) tested directly if a quantitative relationship exists between the number of appropriate Ig VκOxJκ5 mRNA secondary structures bearing WA target sites for the ADAR1 RNA editor (adenosine to inosine, A-to-I) and the recorded incidence, across the full length of the *in vivo* mutated VκOx1Jκ5 sequence, of A-to-G mutations (the standard proxy for A-to-I RNA editing, where W = A or T). We showed that a highly *significant* and *specific* correlation (*P* < 0.002) existed between the frequency (or number) of WA-to-WG mutations and the number of mRNA hairpins that could potentially form at such WA mutation sites. This is still the best direct data-driven evidence for an RNA intermediary in Ig SHM as it implies a direct role for both RNA editing and reverse transcription during SHM *in vivo*, occurring at the highest frequency in the nascent RNA stem-loops presenting WA-sites in dsRNA substrates just emergent from the Transcription Bubble. We now also know that *both* the RNA and DNA moieties in the RNA:DNA hybrid in the Transcription Bubble can potentially be A-to-I edited and contribute to A-to-G and T-to-C somatic mutations (14, 15).

These two sets of positive results consistent with the RNA/RT-based model are completely outside the ambit of the “DNA Deamination Model” neither explained by it nor predicted by it (9, 11). This fact was pointed out explicitly in 2008 (71).

The reader is referred to the considerable detail reviewed in Steele (9, 11) and Lindley and Steele (10), but attention should also be drawn to an awkward fact that cannot be explained by the “DNA Deamination” model yet is readily explained and predicted by the RNA/RT-mechanism (Figure 1)—these are the clear strand biases of somatic mutations whereby mutations off A exceed mutations off T (A >> T, mainly A-to-G >> T-to-C) and yet paradoxically in the same data set or experiment, somatic mutations off G exceed mutations of C (G >> C, mainly G-to-A >> C-to-T). We have illustrated the contradictions of this paradox clearly in Lindley and Steele (10)—as these characteristic strand biases are noted not only in Ig SHM datasets but also in AID/APOBEC driven “Ig-SHM-like responses” in cancer genomes (10, 16).

The other foundation inspiration for our work is the series of discoveries, begun in the 1950s (72, 73), which led to the demonstration in 1970 of reverse transcription in RNA tumor viruses by Howard Temin and David Baltimore (74, 75).

In summary, the DNA-based model of Neuberger and Gearhart, or the “DNA Deamination Model,” is based on AID-induced C-to-U lesions and short-patch error-prone DNA repair by DNA Polymerase-η operating around such lesions (1, 61, 65). However, the RNA/RT-based mechanism (“Reverse Transcriptase Model”) actually subsumes this initiating AID-mediated step and then couples it in the production of the full spectrum of strand-biased mutations at both G:C and A:T base pairs: error-prone cDNA synthesis via an RNA-dependent DNA polymerase (Pol-η) copying the base-modified Ig pre-mRNA template and leading to this now error-filled cDNA copy being integrated back into the normal chromosomal site (Figure 1). The modern form of this mechanism thus depends both on initiating AID C-to-U lesions in DNA and then long-tract error-prone cDNA synthesis of the TS by DNA Polymerase-η acting in its reverse transcriptase mode (59). There are several possible tests. The first could involve measuring the outcome of ADAR A-to-I editing of the RNA and DNA moieties at RNA:DNA hybrids (15) during SHM *in vivo*. Thus on a DNA polymerase-η deficient background (52, 63) the lowered number of mutations at A:T base pairs may allow A-to-I editing of the RNA:DNA hybrid and nascent dsRNA stem loops (Figure 1), but the lack of a RNA-to-DNA copying step could show that T-to-C mutations now balance or exceed A-to-G mutations. Furthermore, a direct test of ADAR deamination in Ig SHM *in vivo* could be achieved in either ADAR1 deficient Aicardi-Goutières Syndrome (AGS) patients (76, 77) or catalytically inactive ADAR1 mouse strains, such as Adar1^E861A/E861A^ Ifih1^−/−^ (78). The caveat to both approaches is a statistically sufficient numbers of A/T mutations and a strategy to avoid or minimize strand bias blunting PCR recombinant artifacts (9).

## Conflict of Interest Statement

The author declares that the research was conducted in the absence of any commercial or financial relationships that could be construed as a potential conflict of interest.

## References

[1] Noia JM Neuberger MS Molecular mechanisms of somatic hypermutation. Annu Rev Biochem (2007) 76:1–22. 10.1146/annurev.biochem.76.061705.090740 17328676

[2] Burnet FM A modification of Jerne’s theory of antibody production using the concept of clonal selection. Aust J Science (1957) 20:67–9. 10.3322/canjclin.26.2.119 816431

[3] Burnet FM The Clonal Selection Theory of Acquired Immunity. Cambridge: Cambridge University Press (1959).

[4] Lederberg J Genes and antibodies. Science (1959) 129:1649–53. 10.1126/science.129.3364.1649 13668512

[5] Cohn M The molecular biology of expectation. In: Plescia OJ Brain W, editors. Nucleic Acids in Immunology. New York: Springer-Verlag NY (1968). p. 671–715.

[6] Weigert MG Cesari IM Yonkovich SJ Cohn M Variability in the Lambda light chain sequences. Nature (1970) 228:1045–7. 10.1038/2281045a0 5483159

[7] Cohn M A rationale for ordering the data on antibody diversity. In: Brent L Holborow J, editors. Progress Immunol. II Biol Aspects I. Amsterdam-Oxford-New York: Nth Holl Publ Co (1974). p. 261–84.

[8] Cunningham AJ The generation of antibody diversity: its dependence on antigenic stimulation. Contemp Top Mol Immunol (1974) 3:1–26. 10.1007/978-1-4684-2838-4_1 4140048

[9] Steele EJ Mechanism of somatic hypermutation: critical analysis of strand biased mutation signatures at A:T and G:C base pairs. Mol Immunol (2009) 46:305–20. 10.1016/j.molimm.2008.10.021 19062097

[10] Lindley RA Steele EJ Critical analysis of strand-biased somatic mutation signatures in TP53 versus Ig genes, in genome-wide data and the etiology of cancer. ISRN Genomics (2013) 2013:18 10.1155/2013/921418

[11] Steele EJ. Somatic hypermutation in immunity and cancer: critical analysis of strand-biased and codon-context mutation signatures. DNA Repair (2016) 45:1–24. 10.1016/j.dnarep.2016.07.001 27449479

[12] Steele EJ Pollard JW Hypothesis: somatic hypermutation by gene conversion via the error-prone DNA->RNA->DNA information loop. Mol Immunol (1987) 24:667–73. 10.1016/0161-5890(87)90049-6 2443841

[13] Steele EJ Lindley RA Somatic mutation patterns in non-lymphoid cancers resemble the strand biased somatic hypermutation spectra of antibody genes. DNA Repair (2010) 9:600–3. 10.1016/j.dnarep.2010.03.007 20418189

[14] Steele EJ Lindley RA. ADAR deaminase A-to-I editing of DNA and RNA moieties of RNA:DNA hybrids has implications for the mechanism of Ig somatic hypermutation. DNA Repair (2017) 55:1–6. 10.1016/j.dnarep.2017.04.004 28482199

[15] Zheng YC Lorenzo C Beal PA. DNA editing in DNA/RNA hybrids by adenosine deaminases that act on RNA. Nucleic Acids Res (2017) 45:3369–77. 10.1093/nar/gkx050 28132026 PMC5389660

[16] Lindley R The importance of condon context for understanding the Ig-like somatic hypermutation starnd-biased patterns in TP53 mutations in breast cancer. Cancer Genet (2013) 206:222–6. 10.1016/j.cancergen.2013.05.016 23880211

[17] Lindley RA Humbert P Larmer C Akmeemana EH Pendlebury CRR. Association between targeted somatic mutation (TSM) signatures and HGS-OvCa progression. Cancer Med (2016) 5:2629–40. 10.1002/cam4.825 27485054 PMC5055158

[18] Blanden RV Rothenfluh HS Zylstra P Weiller GF Steele EJ. The signature of somatic hypermutation appears to be written into the germline IgV segment repertoire. Immunol Rev (1998) 162:117–32. 10.1111/j.1600-065X.1998.tb01435.x 9602358

[19] Steele EJ Lloyd SS Soma-to-germline feedback is implied by the extreme polymorphism at IGHV relative to MHC. Bioessays (2015) 37:557–69. 10.1002/bies.201400213 25810320

[20] Kidd MJ Chen Z Wang Y Jackson KJ Zhang L Boyd SD The inference of phased haplotypes for the immunoglobulin H chain V region gene loci by analysis of VDJ rearrangements. J Immunol (2012) 188:1333–40. 10.4049/jimmunol.1102097 22205028 PMC4734744

[21] Watson CT Steinberg KM Huddleston J Warren RL Malig M Schein J Complete haplotype sequence of the human immunoglobulin heavy- chain variable, diversity, and joining genes and characterization of allelic and copy-number variation. Am J Hum Genet (2013) 92:530–46. 10.1016/j.ajhg.2013.03.004 23541343 PMC3617388

[22] Fleischman JB Pain RH RR Porter RR Reduction of gamma-globulins. Arch Biochem Biophys (1962) Suppl 1:174–80. 13945475

[23] Brenner S Milstein C Origin of antibody variation. Nature (1966) 211:242–6. 10.1038/211242a0 16982853

[24] Smithies O Antibody variability. Somatic recombination between the elements if “antibody gene pairs” may explain antibody variability. Science (1967) 157:267–73. 10.1126/science.157.3786.267 4165728

[25] Edelman GM Gally JA Somatic recombination of duplicated genes: an hypothesis on the origin Ab diversity. Proc Natl Acad Sci U S A (1967) 57:353–8. 10.1073/pnas.57.2.353 16591477 PMC335513

[26] Wu TT Kabat EA An analysis of the sequences of the variable regions of Bence Jones proteins and myeloma light chains and their implications for antibody complimentarity. J Exp Med (1970) 132:211–50. 10.1084/jem.132.2.211 5508247 PMC2138737

[27] Tonegawa S Steinberg C Too many chains – too few genes. In: Cunningham AJ, editor. Generation of Antibody Diversity: A New Look. New York: Academic Press (1976). p. 175–82.

[28] Tonegawa S Hozumai N Matthyssens GH Schuller R Somatic changes in the context and content of immunoglobulin genes. Cold Spring Harb Symp Quant Biol (1977) 41(Pt 2):872–89. 10.1101/SQB.1977.041.01.097 408084

[29] Gearhart PJ Johnson ND Douglas R Hood L IgG Antibodies to phosphorylcholine exhibits more diversity than their IgM counterparts. Nature (1981) 291:29–34. 10.1038/291029a0 7231520

[30] Bothwell ALM Pashkind M Reth M Imanishi-Kari T Rajewsky T Baltimore D Heavy chain variable region contribution to the NPb family of antibodies: somatic mutation evident in a IgG2a variable region. Cell (1981) 24:625–37. 10.1016/0092-8674(81)90089-1 6788376

[31] Selsing E Storb U Somatic mutation of immunoglobulin light-chain variable region genes. Cell (1981) 25:47–58. 10.1016/0092-8674(81)90230-0 6791832

[32] Gearhart PJ. Generation of immunoglobulin variable gene diversity. Immunol Today (1982) 3:107–12. 10.1016/S0167-5699(82)80026-1 25291455

[33] Gearhart PJ Bogenhagen DF Clusters of point mutations are found exclusively around rearranged antibody variable gene. Proc Natl Acad Sci U S A (1983) 80:3439–43. 10.1073/pnas.80.11.3439 6222379 PMC394059

[34] Berek C Griffith GM Milstein C. Molecular events during maturation of the immune response to oxazolone. Nature (1985) 316:412–8. 10.1038/316412a0 3927173

[35] Cumano A Rajewsky K. Clonal recruitment and somatic mutation in the generation of immunological memory to the hapten NP. EMBO J (1986) 5:2459–68. 2430792 10.1002/j.1460-2075.1986.tb04522.x PMC1167140

[36] Golding GB Gearhart PJ Glickman RW. Patterns of somatic mutations in immunoglobulin variable genes. Genetics (1987) 115:169–76. 3557109 10.1093/genetics/115.1.169 PMC1203053

[37] Both GW Taylor L Pollard JW Steele EJ. Distribution of mutations around rearranged heavy-chain antibody variable-region genes. Mol Cell Biol (1990) 10:5187–96. 10.1128/MCB.10.10.5187 2118991 PMC361197

[38] Lebecque SG Gearhart PJ Boundaries of somatic mutation in rearranged immunoglobulin genes: 5′ boundary is near the promoter, 3′ boundary is approximately 1 kb from V-D-J gene. J Exp Med (1990) 172:1717–27. 10.1084/jem.172.6.1717 2258702 PMC2188766

[39] Rogozin IB Solovyov VV Kolchanov NA. Somatic hypermutagenesis in immunoglobulin genes. I. Correlation between somatic mutations and repeats. Somatic mutation properties and clonal selection. Biochim Biophys Acta (1991) 1089:175–82. 10.1016/0167-4781(91)90005-7 2054380

[40] Rogozin IB Sredneva NE Kolchanov NA. Somatic hypermutagenesis in immunoglobulin genes. III. Somatic mutations in the chicken light chain locus. Biochim Biophys Acta (1996) 1306:171–8. 10.1016/0167-4781(95)00241-3 8634334

[41] Steele EJ Rothenfluh HS Both GW. Defining the nucleic acid substrate for somatic hypermutation. Immunol Cell Biol (1992) 70:129–44. 10.1038/icb.1992.18 1398773

[42] Betz AG Neuberger MS Milstein C. Discriminating intrinsic and antigen-selected mutational hotspots in immunoglobulin V genes. Immunol Today (1993) 14:405–11. 10.1016/0167-5699(93)90144-A 8397780

[43] Yelamos J Klix N Goyenechea B Lozano F Chui YL Gonzalez Fernandez A Targeting of non-Ig sequences in place of the V segment by somatic hypermutation. Nature (1995) 376:225–9. 10.1038/376225a0 7617031

[44] Peters A Storb U. Somatic hypermutation of immunoglobulin genes is linked to transcription initiation. Immunity (1996) 4:57–65. 10.1016/S1074-7613(00)80298-8 8574852

[45] Milstein C Neuberger MS Staden R. Both DNA strands of antibody genes are hypermutation targets. Proc Natl Acad Sci U S A (1998) 95:8791–4. 10.1073/pnas.95.15.8791 9671757 PMC21155

[46] Fukita Y Jacobs H Rajewsky K. Somatic hypermutation in the heavy chain locus correlates with transcription. Immunity (1998) 9:105–14. 10.1016/S1074-7613(00)80592-0 9697840

[47] Rada C Ehrenstein MR Neuberger MS Milstein C. Hot spot focusing of somatic hypermutation in MSH2-deficient mice suggests two stages of mutational targeting. Immunity (1998) 9:135–41. 10.1016/S1074-7613(00)80595-6 9697843

[48] Goodman MF. Error-prone repair DNA polymerases in prokaryotes and eukaryotes. Annu Rev Biochem (2002) 71:17–50. 10.1146/annurev.biochem.71.083101.124707 12045089

[49] Muramatsu M Kinoshita K Fagarasan S Yamada S Shinkai Y Honjo T Class switch recombination and hypermutation require activation-induced cytidine deaminase (AID), a potential RNA editing enzyme. Cell (2000) 102:553–63. 10.1016/S0092-8674(00)00078-7 11007474

[50] Rogozin IB Pavlov YI Bebenek K Matsuda T Kunkel TA. Somatic mutation hotspots correlate with DNA polymerase eta error spectrum. Nat Immunol (2001) 2:530–6. 10.1038/88732 11376340

[51] Pavlov YI Rogozin IB Galkin AP Aksenova AV Hanaoka F Rada C Correlation of somatic hypermutation specificity and A-T base pair substitution errors by DNA polymerase-eta during copying of a mouse immunoglobulin kappa light chain transgene. Proc Natl Acad Sci U S A (2002) 99:9954–9. 10.1073/pnas.152126799 12119399 PMC126606

[52] Zeng X Winter DB Kasmer C Kraemer KH Lehmann AR Gearhart PJ DNA polymerase-eta is an A-T mutator in somatic hypermutation of immunoglobulin variable genes. Nat Immunol (2001) 2:537–41. 10.1038/88740 11376341

[53] Bransteitter R Pham P Scharff MD Goodman MF Activation-induced cytidine deaminase deaminates deoxcytidine on single-stranded DNA but requires the action of RNase. Proc Natl Acad Sci U S A (2003) 100:4102–7. 10.1073/pnas.0730835100 12651944 PMC153055

[54] Chaudhuri J Tian M Khuong C Chua K Pinaud E Alt FW. Transcription-targeted DNA deamination by the AID antibody diversification enzyme. Nature (2003) 422:726–30. 10.1038/nature01574 12692563

[55] Dickerson SK Market E Besmer E Papvasiliou FN. AID mediates hypermutation by deaminating single stranded DNA. J Exp Med (2003) 197:1291–6. 10.1084/jem.20030481 12756266 PMC2193777

[56] Chaudhuri J Khuong C Alt FW. Replication protein A interacts with AID to promote deamination of somatic hypermutation targets. Nature (2004) 430:992–8. 10.1038/nature02821 15273694

[57] Shen HM Storb U. Activation-induced cytidine deaminase (AID) can target both DNA strands when the DNA is supercoiled. Proc Natl Acad Sci U S A (2004) 101:12997–3002. 10.1073/pnas.0404974101 15328407 PMC516507

[58] Rada C Di Noia JM Neuberger MS. Mismatch recognition and uracil excision provide complementary paths to both Ig switching and the A/T-focused phase of somatic mutation. Mol Cell (2004) 16:163–71. 10.1016/j.molcel.2004.10.011 15494304

[59] Franklin A Milburn PJ Blanden RV Steele EJ. Human DNA polymerase-eta an A-T mutator in somatic hypermutation of rearranged immunoglobulin genes, is a reverse transcriptase. Immunol Cell Biol (2004) 82:219–25. 10.1046/j.0818-9641.2004.01221.x 15061777

[60] Steele EJ Franklin A Blanden RV Genesis of the strand biased signature in somatic hypermutation of rearranged immunoglobulin variable genes. Immunol Cell Biol (2004) 82:208–18. 10.1046/j.0818-9641.2004.01224.x 15061776

[61] Wilson TM Vaisman A Martomo SA Sullivan P Lan L Hanaoka F MSH2-MSH6 stimulates DNA polymerase eta, suggesting a role for A:T mutations in antibody genes. J Exp Med (2005) 201:637–45. 10.1084/jem.20042066 15710654 PMC2213055

[62] Steele EJ Lindley RA Wen J Weiler GF. Computational analyses show A-to-G mutations correlate with nascent mRNA hairpins at somatic hypermutation hotspots. DNA Repair (2006) 5:1346–63. 10.1016/j.dnarep.2006.06.002 16884961

[63] Delbos F Aoufouchi S Faili A Weill J-C Reynaud C-A. DNA polymerase eta is the sole contributor of A/T modifications during immunoglobulin gene hypermutation in the mouse. J Exp Med (2007) 204:17–23. 10.1084/jem.20062131 17190840 PMC2118439

[64] Basu U Meng FL Keim C Grinstein V Pefanis E Eccleston J The RNA exosome targets the AID cytidine deaminase to both strands of transcribed duplex DNA substrates. Cell (2011) 144:353–63. 10.1016/j.cell.2011.01.001 21255825 PMC3065114

[65] Maul RW Saribasak H Martomo SA McClure RL Yang W Vaisman A Uracil residues dependent on the deaminase AID in immunoglobulin gene variable and switch regions. Nat Immunol (2011) 12:70–6. 10.1038/ni.1970 21151102 PMC3653439

[66] Luan DD Korman MH Jakubczak JL Eichbush TH. Reverse transcription of R2B mRNA is primed by a nick at the chromosomal target site: a mechanism for non-LTR retrotransposition. Cell (1993) 72:595–605. 10.1016/0092-8674(93)90078-5 7679954

[67] Kuraoka I Endou M Yamaguchi Y Wada Y Handa H Tanaka K Effects of endogenous DNA base lesions on transcription elongation by mammalian RNA polymerase II. J Biol Chem (2003) 278:7294–9. 10.1074/jbc.M208102200 12466278

[68] MacPhee DG. Mismatch repair, somatic mutations and the origins of cancer. Cancer Res (1995) 55:5489–92. 7585619

[69] Sack SZ Bardwell PD Scharff MD Testing the reverse transcriptase model of somatic mutation. Mol Immunol (2001) 38:303–11. 10.1016/S0161-5890(01)00058-X 11566323

[70] Steele EJ DNA polymerase-η as a reverse transcriptase: implications for mechanisms of hypermutation in innate anti-retroviral defences and antibody SHM systems. DNA Repair (2004) 3:687–92. 10.1016/j.dnarep.2004.03.040 15177177

[71] Steele EJ. Reflections on the state of play in somatic hypermutation. Mol Immunol (2008) 45:2723–6. 10.1016/j.molimm.2008.02.002 18359085

[72] Temin HM Malignant transformation of cells by viruses. Perspect Biol Med (1970) 14:11–26. 10.1353/pbm.1970.0006 5490760

[73] Temin HM The protovitus hypothesis: speculations on the significance of RNA directed DNA synthesis for normal devlopment and for carcinogenesis. J Natl Cancer Inst (1971) 46:3–7. 5115908

[74] Temin HM Mizutani S RNA-dependent DNA polyerase in virions of Rous Sarcoma Virus. Nature (1970) 226:1211–3. 10.1038/2261211a0 4316301

[75] Baltimore D RNA-dependent DNA polymerase in in virions of RNA tumor virus. Nature (1970) 226:1209–11. 4316300 10.1038/2261209a0

[76] O’Connell MA Mannion NM Keegan LP. The epitranscriptome and innate immunity. PLoS Genet (2015) 11(12):e1005687. 10.1371/journal.pgen.1005687 26658668 PMC4675516

[77] Rice GI Kasher PR Forte GM Mannion NM Greenwood SM Szynkiewicz M Mutations in ADAR1 cause Aicardi-Goutières syndrome associated with a type I interferon signature. Nat Genet (2012) 44:1243–8. 10.1038/ng.2414 23001123 PMC4154508

[78] Liddicoat BJ Piskol R Chalk AM Ramaswami G Higuchi M Hartner JC RNA editing by ADAR1 prevents MDA5 sensing of endogenous dsRNA as nonself. Science (2015) 349:1115–20. 10.1126/science.aac7049 26275108 PMC5444807

